# Deletion of TRPC6 Attenuates NMDA Receptor-Mediated Ca^2+^ Entry and Ca^2+^-Induced Neurotoxicity Following Cerebral Ischemia and Oxygen-Glucose Deprivation

**DOI:** 10.3389/fnins.2017.00138

**Published:** 2017-03-28

**Authors:** Jin Chen, Zhaozhong Li, Jeffery T. Hatcher, Qing-Hui Chen, Li Chen, Robert D. Wurster, Sic L. Chan, Zixi Cheng

**Affiliations:** ^1^Division of Neuroscience, Burnett School of Biomedical Sciences, College of Medicine, University of Central FloridaOrlando, FL, USA; ^2^Department of Kinesiology and Integrative Physiology, Michigan Technological UniversityHoughton, MI, USA; ^3^Department of Clinical Laboratory, The First Central Hospital of TianjinTianjin, China; ^4^Department of Cellular and Molecular Physiology, Stritch School of Medicine, Loyola UniversityMaywood, IL, USA; ^5^Division of Metabolic and Cardiovascular Sciences, Burnett School of Biomedical Sciences, College of Medicine, University of Central FloridaOrlando, FL, USA

**Keywords:** TRPC6, NMDA, Ca^2+^, neurotoxicity, ischemia, oxygen-glucose deprivation (OGD)

## Abstract

Transient receptor potential canonical 6 (TRPC6) channels are permeable to Na^+^ and Ca^2+^ and are widely expressed in the brain. In this study, the role of TRPC6 was investigated following ischemia/reperfusion (I/R) and oxygen-glucose deprivation (OGD). We found that TRPC6 expression was increased in wild-type (WT) mice cortical neurons following I/R and in primary neurons with OGD, and that deletion of TRPC6 reduced the I/R-induced brain infarct in mice and the OGD- /neurotoxin-induced neuronal death. Using live-cell imaging to examine intracellular Ca^2+^ levels ([Ca^2+^]_*i*_), we found that OGD induced a significant higher increase in glutamate-evoked Ca^2+^ influx compared to untreated control and such an increase was reduced by TRPC6 deletion. Enhancement of TRPC6 expression using AdCMV-TRPC6-GFP infection in WT neurons increased [Ca^2+^]_*i*_ in response to glutamate application compared to AdCMV-GFP control. Inhibition of N-methyl-d-aspartic acid receptor (NMDAR) with MK801 decreased TRPC6-dependent increase of [Ca^2+^]_*i*_ in TRPC6 infected cells, indicating that such a Ca^2+^ influx was NMDAR dependent. Furthermore, TRPC6-dependent Ca^2+^ influx was blunted by blockade of Na^+^ entry in TRPC6 infected cells. Finally, OGD-enhanced Ca^2+^ influx was reduced, but not completely blocked, in the presence of voltage-dependent Na^+^ channel blocker tetrodotoxin (TTX) and dl-α-amino-3-hydroxy-5-methyl-4-isoxazole propionic acid (AMPA) blocker CNQX. Altogether, we concluded that I/R-induced brain damage was, in part, due to upregulation of TRPC6 in cortical neurons. We postulate that overexpression of TRPC6 following I/R may induce neuronal death partially through TRPC6-dependent Na^+^ entry which activated NMDAR, thus leading to a damaging Ca^2+^ overload. These findings may provide a potential target for future intervention in stroke-induced brain damage.

## Introduction

Ischemic stroke is a devastating disease that is one of the leading causes of death. A critical deficit of cerebral blood flow (CBF) during stroke rapidly triggers neuronal depolarization that releases a large amount of glutamate into the extracellular space (Drejer et al., 1985; Choi et al., 1990; Choi, 1992). Glutamate binds to and activates dl-α-amino-3-hydroxy-5-methyl-4-isoxazole propionic acid (AMPA) receptors and N-methyl-d-aspartic acid (NMDA) receptors (McLennan, 1983; Choi, 1988a,b). Overactivation of the AMPA and NMDA receptors opens their associated ion channels to produce an increased influx of Na^+^ and Ca^2+^, leading to an ionic imbalance within neurons (Choi, 1993; Arundine and Tymianski, 2004). Ca^2+^ overload has been shown to be related to Na^+^ entry (Yu and Salter, 1998; Yu, 2006). It is well established that the loss of homeostasis of Na^+^ and Ca^2+^ plays an important role in ischemia-induced neuronal damage (Breder et al., 2000; Bano and Nicotera, 2007). Thus, over-stimulation of glutamate receptors can be considered as a primary intracellular event that induces neuronal death in stroke. Likewise, upon oxygen and glucose deprivation (OGD) which is a well-established *in vitro* model of ischemia (Tasca et al., 2015), glutamate is massively released by neurons, and the resulting increase in the intracellular Ca^2+^ concentration ([Ca^2+^]i) causes a delayed neuronal cell death (Caldeira et al., 2014). Therefore, treatments which blocks glutamate-ionotropic receptors can protect neurons from ischemic insult and reduce brain infarction in animals (Choi et al., 1988; Chen et al., 1992). Similarly, Ca^2+^ uptake and neuronal cell death following OGD can also be prevented by the NMDA receptor (NMDAR) antagonist (Goldberg and Choi, 1993). Though the blockade of glutamate receptors shows dramatic neuroprotective effects in animal stroke and *in vitro* OGD models, clinical trials aimed at reducing ischemic brain injury by targeting NMDA and AMPA receptors, failed to achieve satisfactory effects (De Keyser et al., 1999; Gladstone et al., 2002; Lo et al., 2003). Therefore, there is a great need to develop new, potential drug targets for the treatment of strokes (Roth and Liesz, 2016).

More recent studies have indicated the existence of other routes of ionic entry and imbalance within the injured neurons, such as involving the transient receptor potential (TRP) family, in particular, the melastatin subfamily of TRP proteins (TRPM). TRPM7 is a well-studied protein among this subfamily which has been shown to be linked with ischemia injured neurons (Aarts and Tymianski, 2005). TRPM7 is a non-selective cation channel that conducts both monovalent ions (Na^+^ and K^+^) and divalent ions (Ca^2+^, Mg^2+^ and other trace metal ions) (Bae and Sun, 2011). TRPM7 channels have been reported as an essential mediator of anoxic neuronal death due to its Ca^2+^-permeable, non-selective cation channel characteristics (Aarts et al., 2003; MacDonald and Jackson, 2007). Suppression of hippocampal TRPM7 channels could prevent neuronal death in brain ischemia (Sun et al., 2009). Carvacrol, acting as a TRPM7 inhibitor, protects brain from neonatal hypoxic-ischemic injury (Chen et al., 2015). Thus, TRPM7 becomes a promising therapeutic target for stroke treatment (Bae and Sun, 2011, 2013).

The other member of the TRP family, canonical subfamily of TRP proteins (TRPC) subfamily is also permeable to Na^+^ and Ca^2+^ non-selectively (Eder et al., 2005; Bush et al., 2006). The TRPC family is comprised of seven different channels (TRPC1-TRPC7) that are broadly expressed (Khairatkar-Joshi et al., 2015; Ong et al., 2016). Among them, several TRPCs (TRPC1, 3, 4, 5, and 6) are expressed in the nervous system and play roles in neuronal growth, path finding, and differentiation (Montell et al., 2002; Riccio et al., 2002). TRPCs play a prominent role in fundamental cellular events ranging from transcriptional regulation to cell proliferation and death (Birnbaumer et al., 1996; Montell et al., 2002). In particular, TRPC6 is a member of the TRPC subfamily. Similar to TRPM7, TRPC6 channels are non-selective cation channels that are permeable to Na^+^ and Ca^2+^. Though many aspects of the physiology and regulation of TRPC6 are still elusive, an increasing number of important pathological conditions are now being linked to TRPC6 dysfunctions (Dietrich and Gudermann, 2014). TRPC6 has been shown to contribute to the sustained Ca^2+^ elevation that is responsible for glioma proliferation (Bomben and Sontheimer, 2008; Ding et al., 2010), hypertrophic growth of cardiomyocytes (Bush et al., 2006; Kuwahara et al., 2006; Onohara et al., 2006; Nishida and Kurose, 2008), proteinuric kidney disease (Möller et al., 2007) and pulmonary hypertension (Lin et al., 2004; Weissmann et al., 2006). Activation of TRPC6 was responsible for lung ischemia-reperfusion induced edema in mice (Weissmann et al., 2012) and high glucose- or oxidative stress-induced podocyte ischemic injury (Yang et al., 2013; Zhao et al., 2015) and downregulation of TRPC6 by Klotho showed cardioprotective effects in the mouse heart (Xie et al., 2012).

TRPC6 channels are localized at the excitatory synapses and may regulate neuronal excitability (Zhou et al., 2008). Activation of TRPC6 channels is not dependent upon depolarization (Montell et al., 2002; Ramsey et al., 2006). Since TRPC6 channels are permeable to Na^+^ and Ca^2+^, we hypothesized that ischemic stroke and OGD may increase TRPC6 expression which may, in turn, activate NMDA receptors (NMDAR) and enhance Ca^2+^ influx. Thus, deletion of TRPC6 would be expected to reduce ischemia-induced brain damage and OGD-/glutamate-induced cortical cell death. Our study has demonstrated that TRPC6-mediated Ca^2+^ influx enhanced excitotoxicity by facilitation of NMDAR activation and TRPC6 deletion attenuated ischemia-induced neuronal death.

## Materials and methods

### Materials

EBSS (Earle's Balanced Salt Solution), Hank's buffer, Glutamax, and penicillin-streptomycin, Trizol, SuperScript II RNaseH reverse transcriptase and T-PER buffer were purchased from Invitrogen. GoTaq Green was obtained from Promega. Neurobasal medium, B27, Dulbecco's Modified Eagle Medium (DMEM), and bovine calf serum were purchased from Hyclone. 2, 3, 5-triphenyltetrazolium chloride (TTC), propidium iodide (PI), protease inhibitor cocktail, N-methyl-D-glucamine (NMDG) and Trypan blue were obtained from Sigma. Other reagents include: CNQX, MK801 (Molecular Probes); tetrodotoxin (TTX, Alamone); Fura-2 acetoxymethyl ester (Fura-2 AM, Molecular Probes). Recombinant adenoviruses AdCMV-GFP and AdCMV-TRPC6-GFP-Myc-tag were kindly provided by Dr. Christopher B. Newgard. The sources of the primary antibodies were as following: TRPC6 (Sigma, Lot# 8831P1), MAP2 (Cell Signaling, #4542), and Beta-Actin (Abcam, ab8227). The goat anti-rabbit and anti-mouse secondary antibodies conjugated to horse radish peroxidase were from Jackson Immunoresearch.

### Animals

Wild type WT mice (C57 BL/6J) were used as the background to generate TRPC6^−/−^ mice. The generation of TRPC6^−/−^ mice has been previously described (Dietrich et al., 2005). The TRPC6^−/−^ mice have been obtained from Dr. Lutz Birnbaumer (National Institute of Environmental Health Sciences) and were housed in in the transgenic animal facility (TAF). One male WT mouse was housed in the same cage with one female WT mouse. Similarly, one male TRPC6^−/−^ mouse was housed in the same cage with one female TRPC6^−/−^ mouse. The pregnant mice were separated from the males and kept in a single cage as soon as they showed signs of pregnancy. The pregnant mice were checked daily. The animal protocol had been approved by UCF IACUC. All experiments were conducted in accordance with the recommendations of UCF IACUC.

### Focal cerebral ischemia model

TRPC6^−/−^ and WT control mice were maintained on a 12 h light/12 h dark cycle with food and water. Three-four month-old male mice were used for this experiment. The focal cerebral ischemia/reperfusion model was generated following that described previously (Culmsee et al., 2001; Arumugam et al., 2006; Wei et al., 2011). Briefly, mice were anesthetized with isofluorane (3–4% mixed with oxygen). The animals were maintained under anesthetic using an anesthesia machine with a scavenger until the completion of the surgery. The depth of anesthesia was assessed by the amplitude of the leg twitch in response to the pinch of the toes. A midline incision was made in the neck, and the left external carotid and pterygopalatine arteries were isolated and ligated with 5-0 silk thread. The internal carotid artery (ICA) was occluded with a small clip applied at a site that was peripheral to the bifurcation of the ICA and the pterygopalatine artery. The common carotid artery (CCA) was ligated with 5-0 silk thread. The external carotid artery (ECA) was cut, and the tip of a 6-0 nylon monofilament was inserted into the ECA. After the clip at the ICA was removed, this nylon thread was advanced into the middle cerebral artery (MCA) until light resistance was felt. The nylon thread and the CCA ligature were removed after 60 min of occlusion to initiate reperfusion. CBF was measured using a laser Doppler perfusion monitor and all CBF measurements were conducted with the mouse fixed in a plastic frame with the probe placed in the region of cerebral cortex perfused by the MCA as previously described (Arumugam et al., 2006). All surgical instruments were autoclaved prior to use. The animals were placed on a heating pad during the surgery and recovery from anesthesia, during which time the animals were be closely monitored. Post-operative pain was relieved using buprenorphine (0.03 mg/kg body weight; IP). Analgesia was given post-op prior recovery from anesthesia and then every 12 h for 2 days. The animals were euthanized at various time points (12, 18, 24, 48, 72 h after the surgery), and the ipsilateral and contralateral cortices of their brains were removed for immunohistochemical and biochemical analyses.

### Quantification of cerebral infarction

After 24 h of reperfusion, the mice were euthanized with isofluorane (5% mixed with oxygen). The brains were immediately removed and placed into ice-cold PBS for 15 min, and then 2-mm coronal sections were cut. The brain sections were stained with TTC (2%) in PBS at 37°C for 15 min. The stained sections were photographed, and the digitized images were used for analysis. The borders of the infarct in each brain slice were outlined, and the area quantified using an ImageJ software. Infarct volume was calculated by integration of infarct areas for all slices of each brain (Arumugam et al., 2006; Tu et al., 2010; Pei et al., 2015).

### Primary cortical neuronal cultures

Primary cultures of mouse cortical neurons were prepared from the 18-day-old fetuses of pregnant female mice as previously described (Brewer et al., 1993; Brewer, 1997; Szychowski et al., 2015). Briefly, brain cortices were harvested by removal of the brainstem and hippocampus followed by mechanical trituration. After the dissected tissue was minced and gently digested with trypsin, the cells were suspended in Neurobasal medium (Gibco) supplemented with 2% B-27 supplement (Invitrogen), 2 mM glutamax, and 1% antibiotic (50 U/mL penicillin, and 0.05 mg/mL streptomycin). This procedure typically yields cell cultures that contain about 90% neurons and 10% astrocytes (Kajta et al., 2004). Live cells were identified using Typan blue staining and counted. The cells were seeded at a density of 5 × 10^5^ cells per well in 6 well plate (all plates were pre-coated with poly-D-lysine) and maintained at 37°C with 5% CO_2_. Half of the medium was changed every 3 days, and the cells were cultured for 14-16 days.

### Oxygen-glucose deprivation

After 14–16 days of culture, the primary cortical neurons were challenged with oxygen-glucose deprivation (OGD) (Goldberg and Choi, 1993). OGD was induced by washing and incubating the cultures in a pre-equilibrated glucose-free balanced salt solution in the Billups-Rothenberg anaerobic chamber containing 95% nitrogen and 5% CO_2_. The chamber was sealed and incubated for 2 h at 37°C. At the end of the procedure, culture medium was removed and replaced with serum-containing Neurobasal medium prior to returning the neuronal cultures to normoxia (95% oxygen and 5% CO_2_) for 24 h. Control cultures were incubated for 2 h in a similar balanced salt solution containing 5.5 mM glucose under normoxic conditions.

### RT-PCR

Total RNA was extracted from the ipsilateral and contralateral cortices of the brain tissue using Trizol reagent following the manufacture's protocol. Reverse transcription was started with 2 μg of total RNA at 42°C for 60 min in 20 μL reaction mixture containing 20 U RNase inhibitor, 0.25 mM of each dNTP, 0.5 μg Oligo(dT)_15_, 200 U SuperScript II RNaseH reverse transcriptase. Synthesized first-strand cDNA was then amplified by PCR in 25 μL reaction volume, containing 5 μL DNA template, 12.5 μL GoTaq Green Master Mix, a pair of primer for TRPC6 and inner control β-actin, respectively (10 pmol per primer). An appropriate cycle number was chosen to avoid the accumulation of by-products and yield accurate replication of target DNA before the characteristic plateau phase was reached in the PCR amplification curve. PCR procedure included: pre-denatured at 95°C for 5 min; amplification at 95°C 50 s, 56°C 1 min, 72°C 1 min; and extended at 72°C for 10 min. Aliquots of the PCR product were separated using agarose gel electrophoresis and visualized with ethidium bromide staining. The following oligonucleotide primers were used: for TRPC6, sense 5′-CAG ATC ATC TCT GAA GGT CTT TAT GC-3′ and anti-sense 5′-TGT GAA TGC TTC ATT CTG TTT GCG CC-3′; for β-actin, sense 5′-TGC TAT CCC TGT ACG CCT CT-3′, anti-sense 5′-GGA GGA GCA ATG ATC TTG A-3′.

### Western blot

Protein from ipsilateral and contralateral cortices was extracted using T-PER tissue protein extraction buffer with protease inhibitor cocktail (Sigma). Protein concentration was determined using the BCA protein assay kit (Pierce, USA). Fifty μg of protein was separated by SDS-PAGE (8–12%) and transferred to nitrocellulose membranes. The membranes were blocked in 5% non-fat milk for 1 h at room temperature, followed by an overnight incubation at 4°C with antibodies raised against TRPC6 or β-actin. Membranes were then washed and incubated with secondary antibodies for 1 h at room temperature. Protein bands were visualized using a chemiluminescence detection kit (Amersham Biosciences).

### Adenoviral transduction

For adenovirus construction, cytomegalovirus (CMV) promoter-driven human Myc-tagged TRPC6 adenovirus was constructed by cloning cDNA constructs into the pAdTrack shuttle vector and using the Ad-Easy system to generate the recombinant adenoviruses as previously described (He et al., 1998; Hayes et al., 2013). Approximately 14–16 days after cortical neuron seeding, the medium was removed and cells were transduced at viral doses of up to 20 pfu/cell for 4 h (Robert et al., 1997). After the virus-containing medium was removed, the fresh medium was added. At 24-h intervals, cells were collected for the experiments.

### Quantification of neuronal death

Propidium iodide (PI) staining of nuclei (red) in primary neurons from WT and TRPC6^−/−^ mice following OGD, 100 μM glutamate or 50 μM NMDA pretreatment were performed. Primary neuron cells were fixed with cold 70% ethanol, permeabilized with 0.5% Triton X-100 in PBS for 5 min and RNase treated (100 U/ ml; Sigma) at room temperature for 120 min. Nuclei were stained with 1 μg/ml propidium iodide (PI; Sigma) for 10 min (Ito et al., 2004). For double-labeling of the nuclei and cell body with dendrites, non-specific sites were blocked with 5% BSA in PBS at room temperature for 60 min, and cells were incubated at 4°C with rabbit MAP2 (Cell Signaling, #4542) overnight after fixation (Soltani et al., 2005; Campbell et al., 2014). The cells were incubated with goat anti-rabbit IgG (H+L), Alexa Fluor® 488 (Invitrogen) for 60 min after washing 3 times. Then, nuclei were stained with 1 μg/ml propidium iodide (PI; Sigma) for 10 min). Coverslips were mounted with mounting medium containing anti-bleaching agent and examined with a fluorescence microscope (Nikon 80i). The number of PI-labeled bright condensed nuclei was counted and expressed as the percentage of the total number of PI positive cells counted, indicating the percent of cell death. All experiments were repeated using at least three independent cultures.

### Intracellular Ca^2+^ imaging

After 14–16 days of culture, these neurons were loaded with the fluorescent dye Fura-2 acetoxymethyl ester (Fura-2 AM) (3 μM) for 30 min in humidified 5% CO_2_ incubator at 37°C, washed 3 times in HEPES-KRH buffer (116 mM NaCl, 4 mM KCl, 1 mM MgCl_2_, 1.8 mM CaCl_2_, 25 Mm Glucose, 10 mM HEPES, pH 7.4) and incubated for additional 30 min. Then, the changes in free intracellular Ca^2+^ concentration ([Ca^2+^]_*i*_) in neurons loaded with Fura-2 were recorded and measured using the Nikon Live Image System with Nikon Elements software (Nis-Elements AK 4.13). Images were taken every 10 s with an exposure time of 200 ms. Fura-2 dual excitation images were captured with a camera (AnDor; Fluor 40× objective). The baseline Fura-2 fluorescence intensity (expressed as the ratio of 340/380 nm) was measured 1 min before glutamate application. Glutamate application (100 μM) at 60 s after initiation of recording was used to evoke Ca^2+^ influx. An increase in relative Fura-2 340/380 fluorescence indicates an increase in free intracellular Ca^2+^ concentration (Hirst et al., 2005; Barreto-Chang and Dolmetsch, 2009). The fluorescence intensity of primary neurons (ratio of 340/380 nm) in each image frame was normalized relative to the background intensity. Appropriate regions of interest, representing a change in Ca^2+^ influx, were selected, and the average ratio of fluorescence was represented in graphs. The change of fluorescence intensity (F) of each neuron at the different time points was normalized relative to the initial intensity (F0) of that neuron at 0 s (before glutamate application). The mean dynamic changes in [Ca^2+^]_*i*_ were determined at each time point for 3–10 neurons in each experiment.

### Statistical analysis

Values were represented as mean ± S.E.M. Student *t*-test was used for comparison between two groups. Statistical comparisons were performed using ANOVA and Newman-Keuls *post-hoc* test for pairwise comparisons. Statistical significance was accepted at *P* < 0.05.

## Results

### Ischemia/reperfusion (I/R) and OGD increased TRPC6 expression

**Figure 1:**
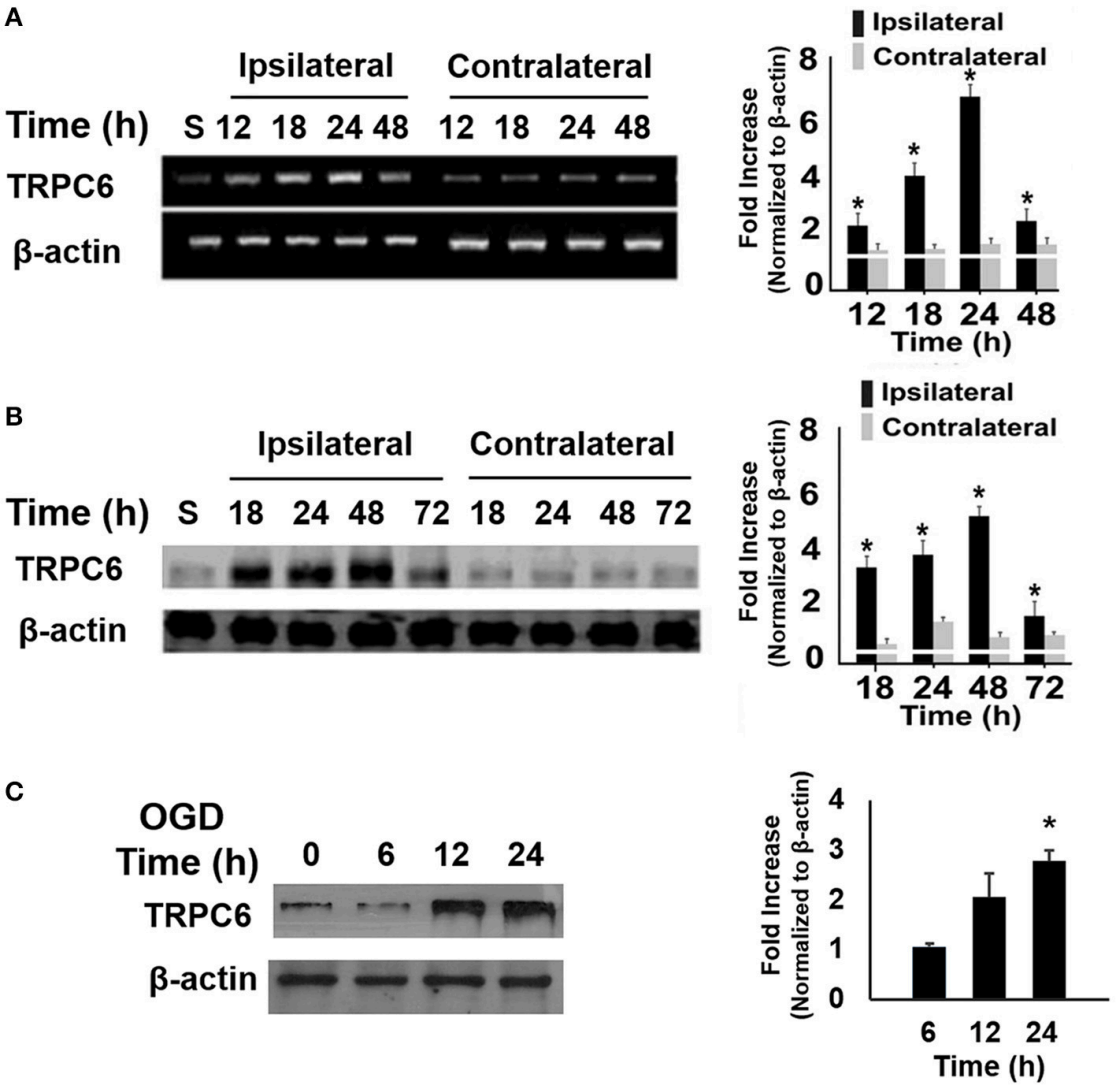
**Ischemia/Reperfusion (I/R) and OGD increased TRPC6 expression. (A,B)** Time course analysis of TRPC6 mRNA **(A)** and protein **(B)** expressions in the ipsilateral and contralateral cortices of mice subjected to I/R. Quantitative analyses of the changes in TRPC6 mRNA and protein levels at the indicated time points after I/R were determined by densitometry. Fold increases are relative to Sham (S). ^*^P < 0.01 compared to corresponding values in the contralateral cortices (*n* = 4/group). **(C)** TRPC 6 protein in WT cortical neurons subjected to OGD. Immunoblotting was performed with 50 μg of total proteins and probed with antibodies to TRPC6. β-actin was used as a loading control. Three independent experiments at each time point (*n* = 3). ^*^P < 0.01, compared with control groups (0 h).

To determine whether ischemia altered the TRPC6 expression level, we measured mRNA levels of TRPC6 in the brains following I/R. We found that mRNA expression of TRPC6 was markedly upregulated, starting at 12 h after I/R in the ipsilateral, but not in the contralateral cortices (Figure 1A). Analogously to its transcript, TRPC6 protein levels were significantly higher in the ipsilateral side than in the contralateral cortices (Figure 1B). TRPC6 protein levels were also measured at 6, 12, and 24 h following OGD in primary cortical neurons. Immunoblot showed a significant increase of the TRPC6 protein level in OGD-treated cortical neurons 24 h after OGD treatment (Figure 1C), that was consistent with the I/R mice as shown in Figure 1B. These results demonstrated that I/R and OGD induced a significant increase in TRPC6 expression in neurons.

### TRPC6 deletion attenuated I/R-induced brain infarct size and protected neurons from OGD-induced cell death

**Figure 2:**
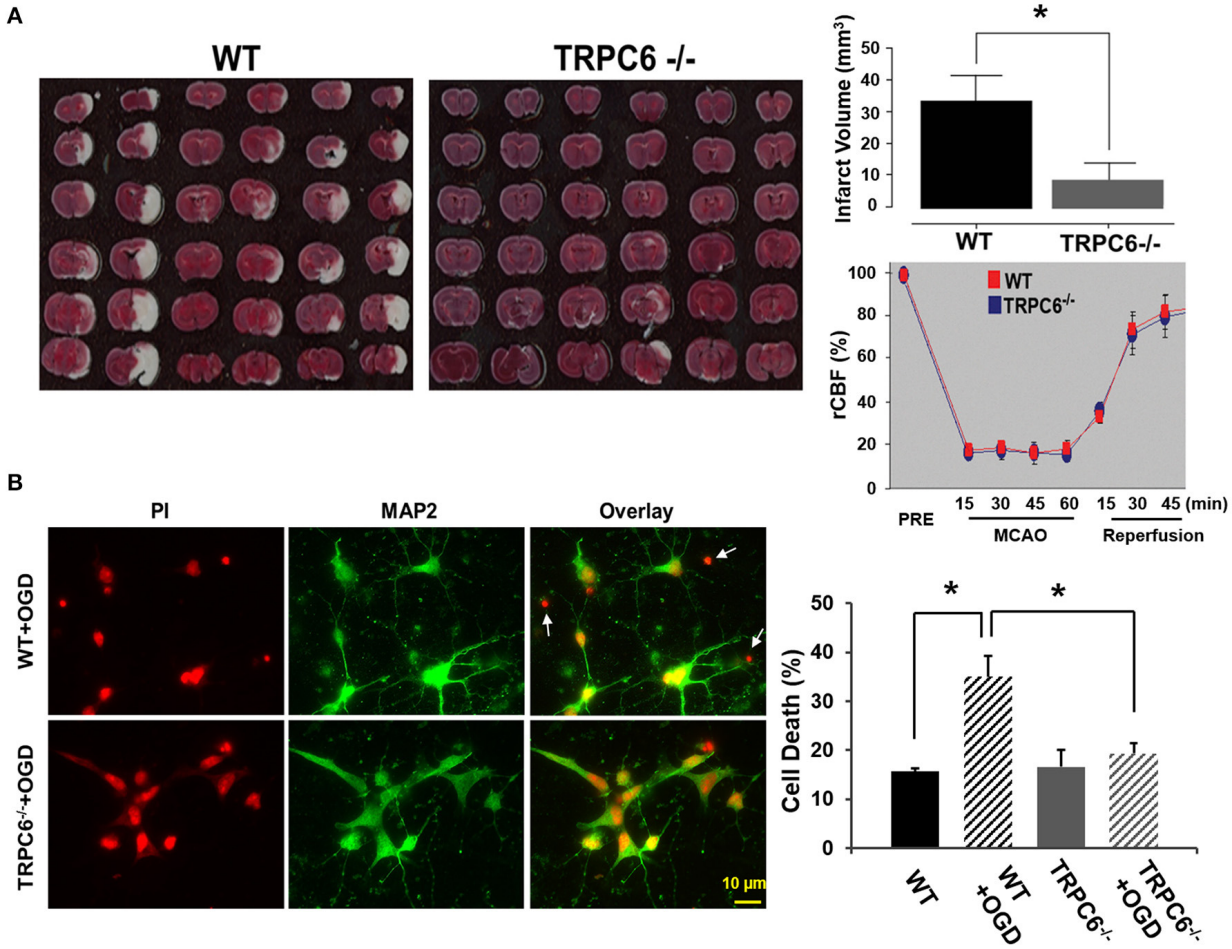
**TRPC6 deletion attenuated I/R-induced brain infarct size and protected neurons from OGD-induced cell death. (A)** Images of the brain sections from WT and TRPC6^−/−^ mice 24 h after a 60 min-MCAO that were stained with TTC. Bar graph shows the infarct volume. Infarct volume was calculated by integration of infarct areas that were not stained by TTC for all slices of each brain. ^*^P < 0.01 (*n* = 6 /group). Regional cerebral blood flow (rCBF) in TRPC6^−/−^ and WT mice at the indicated time points before (PRE), during 60 min MCAO and 45 min reperfusion (*n* = 3–4/group). rCBF was measured over the MCA territory by laser Doppler flowmetry. Baseline rCBF measured before MCA occlusion was defined to be 100% flow. **(B)** Propidium iodide (PI) staining of nuclei and cell bodies (red) and immunocytochemical labeling of MAP2 (green) of cell body and dendrites in primary neurons from WT and TRPC6^−/−^ mice following OGD pretreatment. Scale bar, 10 μm. The small, bright condensed nuclei (<2 μm in diameter) without stomata and dendrites indicate dead neurons as indicated by arrows. Bar graph shows the percentage of cell death in WT and TRPC6^−/−^ primary neurons with or without OGD pretreatment ^*^P < 0.01 (*n* = 3 independent experiments/group). In each experiment, three wells and >100 neurons/well were counted.

To determine whether TRPC6 plays a role in the brain infarct size, we employed the I/R model in WT and TRPC6^−/−^ mice. Two days after I/R, we measured the amount of the brain damage. We found that the infarct size was significantly decreased in the TRPC6^−/−^ mice compared to WT mice (Figure 2A), indicating that TRPC6 deletion rendered the brain less susceptible to the ischemic insults. To determine whether TRPC6 ablation may alter physiologic variables that can influence the function outcome in ischemic stroke, regional cerebral blood flow (rCBF) was measured using a laser Doppler probe as previously described (Arumugam et al., 2006). rCBF in the area blood-supplied by MCA was reduced equivalently to near constant values, averaging approximately 20% of normal flow. Upon reperfusion, rCBF recovered both in WT and TRPC6^−/−^ mice. Hence, TRPC6 ablation does not affect rCBF before, during, or after ischemia (Figure 2A).

To examine whether TRPC6 plays a role in OGD-induced neuronal cell death, we challenged WT and TRPC6^−/−^ cortical neurons with OGD. Cell viability was assessed by PI staining (red) that had bound to the nucleotide. The dying neurons had the smaller size of nuclei (<2 μM in diameter) than the other neurons that have the normal size of nuclei (4–5 μM) (Hezel et al., 2012). Compared to WT neurons challenged with OGD, there were significantly fewer condensed PI-labeled nuclei in TRPC6^−/−^ neurons in the same condition (Figure 2B, Left, Red). To further identify neurons, we used the neuron-specific marker MAP2 to label cell body and dendrites (Figure 2B, Left, Green). PI and MAP2 double-labeling of neurons with OGD pretreatment were shown in Figure 2B, Left. These data showed that deletion of TPRC6 significantly reduced the OGD-induced neuronal cell death (as indicated by the bright shrinking nuclei and lack of MAP2).

### TRPC6^−/−^ protected against glutamate or NMDA-induced cell death

**Figure 3:**
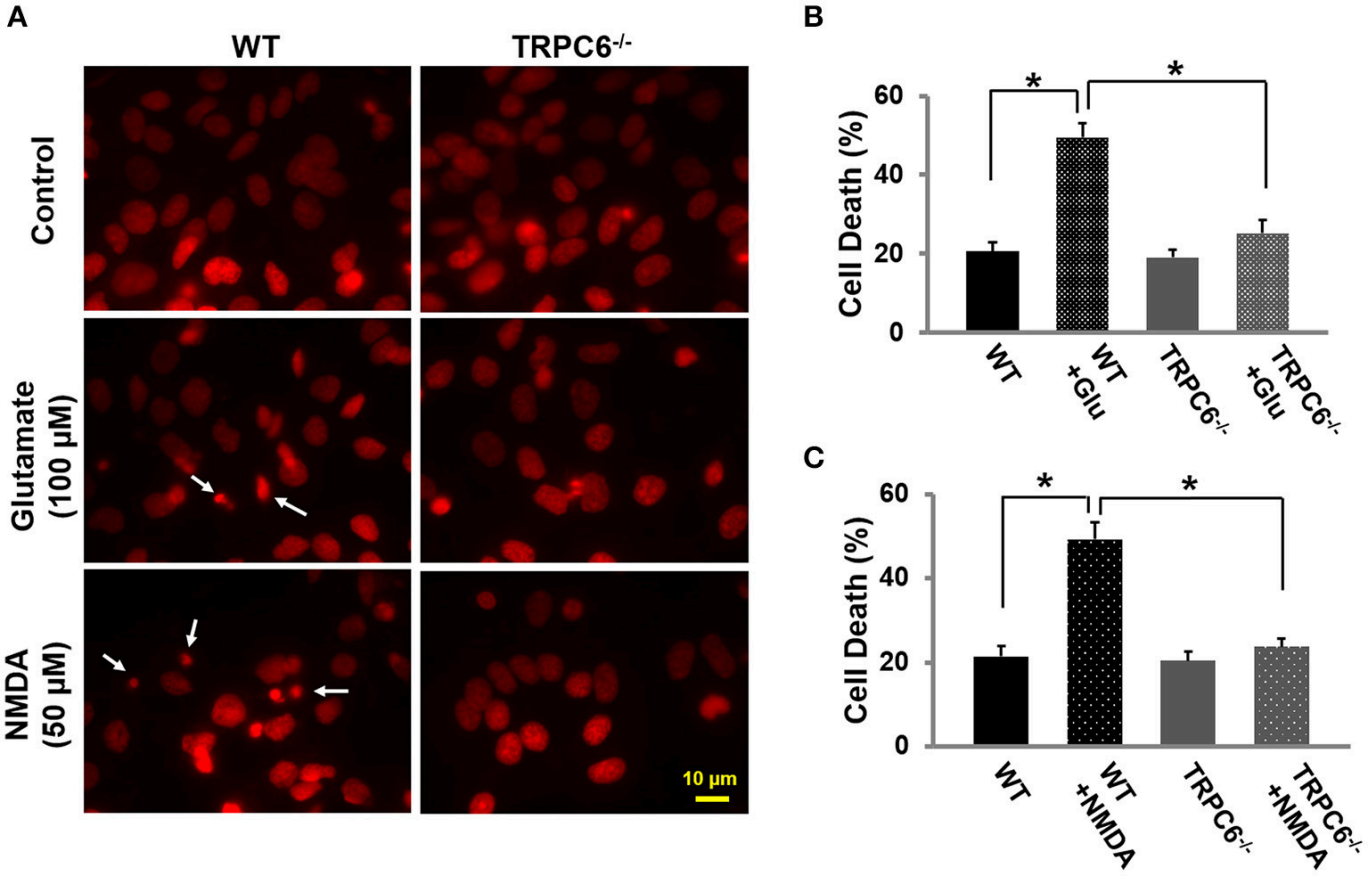
**TRPC6 deletion protected primary cortical neurons from glutamate- or NMDA-induced neuronal death.(A)** Representative images using propidium iodide (PI) staining in WT and TRPC6^−/−^ primary neurons with glutamate (100 μM) or NMDA (50 μM) pretreatments, respectively. The small, bright condensed nuclei (<2 μm in diameter) (arrow) show the dying neurons. Scale bar, 10 μm. **(B,C)** Cell death after glutamate and NMDA pretreatment in WT and TRPC6^−/−^ neurons. ^*^P < 0.01 (*n* = 3 independent experiments/group). In each experiment, three wells and >100 neurons/well were counted.

To evaluate the role of TRPC6 in excitotoxic neuronal death, we treated WT and TRPC6^−/−^ cortical neuron cultures (14–16 days*)* with glutamate (100 μM) and NMDA (50 μM) for 30 min, respectively. Twenty-four hours after glutamate or NMDA treatment, the cultured neurons were fixed and cell viability was assessed by PI staining (red). These results showed that glutamate and NMDA significantly increased neuronal cell death compared to normal untreated neurons. Deletion of TRPC6 significantly protected neurons from glutamate and NMDA-induced cell death (Figures 3A–C).

### OGD increased Ca^2+^ influx in response to glutamate and TRPC6 deletion decreased OGD-induced Ca^2+^ overload

**Figure 4:**
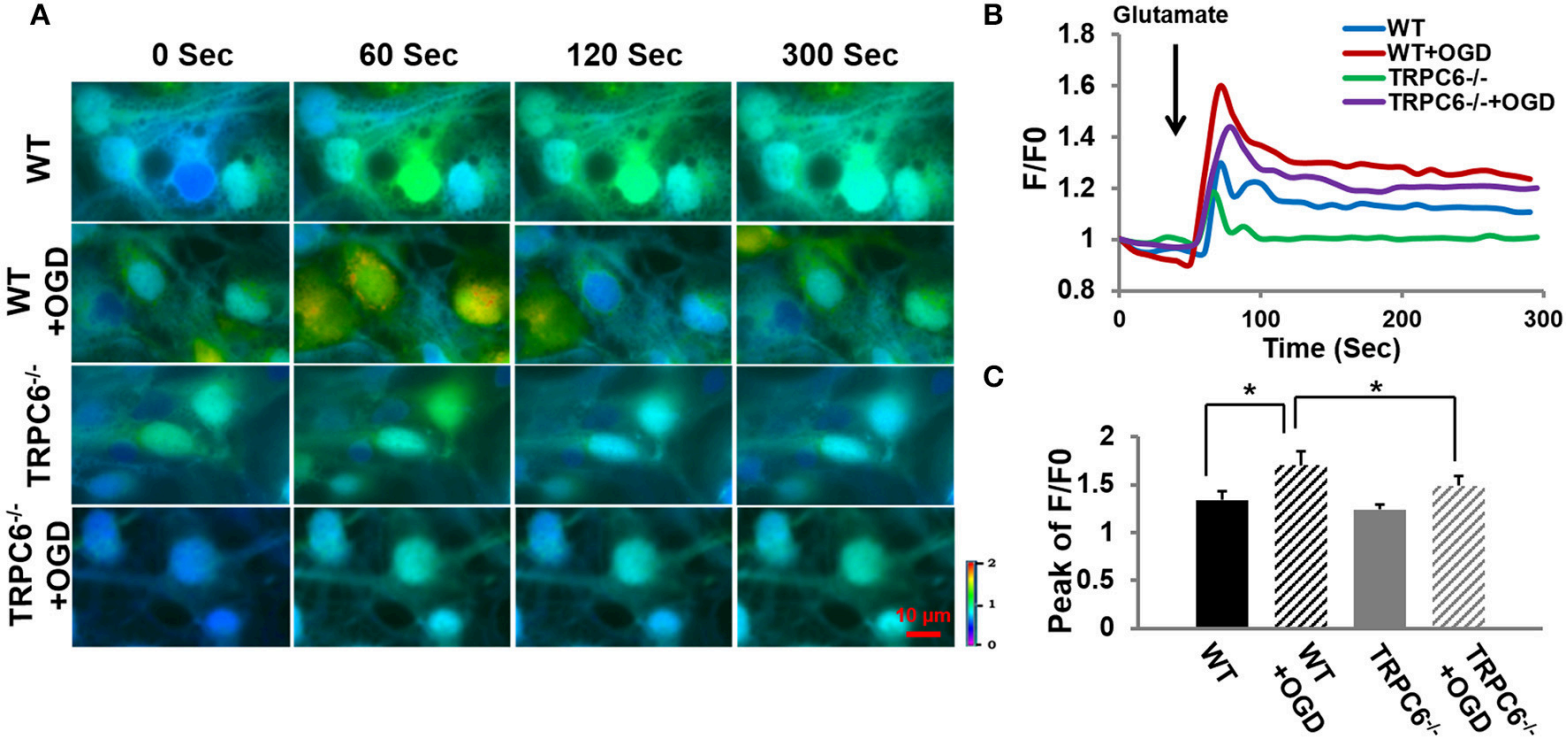
**TRPC6 deletion decreased OGD-induced Ca^**2+**^ overload. (A)** Ratiometric fluorescence images of Fura-2 loaded WT and TRPC6^−/−^ primary neurons in response to glutamate (100 μM) application with and without OGD pretreatments. Images were taken every 10 s during the recording. Intracellular [Ca^2+^]i intensity is indicated by the ratio of 340/380 nm and encoded by pseudocolor as shown in the color bar. **(B)** Traces show the time course of [Ca^2+^]i which was analyzed using the Nikon live cell imaging software. Glutamate was added as indicated by an arrow before 60 s. Ten neurons were sampled and analyzed in each experiment. F/F0 is expressed as the Fura-2 fluorescence ratio 340/380 nm (F) relative to the initial fluorescence ratio (F0) at each time point. At time 0, F/F0 is 1. **(C)** Bar graph shows the mean peaks of F/F0 traces of the 4 groups. ^*^P < 0.01 (*n* = 3 independent experiments/group).

Because Ca^2+^ overload is one of the major contributors to OGD-induced neuronal cell death (Goldberg and Choi, 1993; Bruno et al., 1994; Honda et al., 1994), we next determined whether TRPC6 deletion protects against OGD -induced Ca^2+^ influx. Figure 4A included original recordings of intracellular Ca^2+^ level [Ca^2+^]_*i*_ at different time frames in response to glutamate application at 60 s after the start of recording (0 s) into the bath. Figures 4B,C showed the time course and the peak of [Ca^2+^]_*i*_ in response to glutamate application, respectively. OGD pretreatment significantly increased [Ca^2+^]_*i*_ in neurons in both WT and TRPC6^−/−^ neurons compared to the [Ca^2+^]_*i*_ level at the start of recording (0 s) where F/F0 equaled 1. Deletion of TRPC6 significantly attenuated OGD-induced [Ca^2+^]_*i*_ increase compared to control (*P* < 0.01). These observations indicate that TRPC6 contributes to OGD-increased Ca^2+^ influx.

### Ca^2+^ influx was enhanced in TRPC6 overexpressed neurons, independent of AMPA receptors

**Figure 5:**
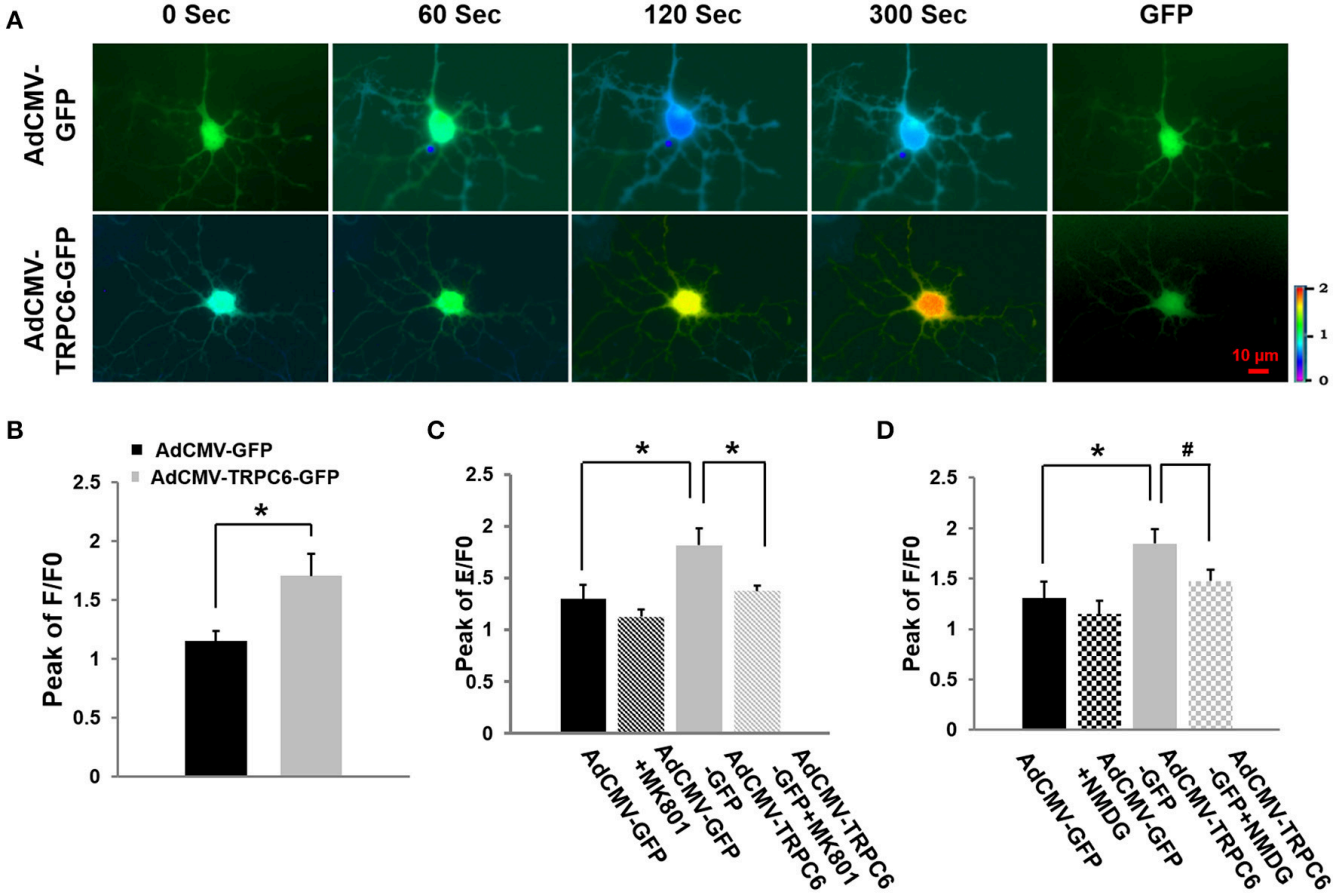
**Overexpression of TRPC6 increased glutamate-evoked Ca^**2+**^ influx in WT cortical neurons: effects of AMPA receptor antagonist, NMDA receptor antagonist, and Na^**+**^-free medium. (A)** AdCMV-GFP and AdCMV-TRPC6-GFP were infected WT neurons with GFP fluorescence which are indicated as GFP in the right column. Ratiometric fluorescence images of Fura-2 AM loaded AdCMV-GFP (control) and AdCMV-TRPC6-GFP (TRPC6 overexpression) neurons after glutamate (100 μM). Images were taken every 10 s during recording. [Ca^2+^]_*i*_ was analyzed using Fura-2 as ratio of 340/380 nm and encoded by pseudocolor as in Figure 4. **(B)** AdCMV-TRPC6-GFP neurons exhibited a significantly higher glutamate-evoked [Ca^2+^]_*i*_increase compared to AdCMV-GFP control neurons in the presence of the AMPA antagonist (10 μM CNQX). The bar graph shows the peak of F/F0 in AdCMV-GFP and AdCMV-TRPC6-GFP neurons after glutamate. Three neurons were collected per experiment. ^*^P < 0.01 (*n* = 3 independent experiments/group). **(C)** TRPC6-mediated Ca^2+^ influx was blocked by an irreversible NMDAR channel blocker MK-801 (20 μM). The bar graph shows the peaks of F/F0 in AdCMV-GFP (control) and AdCMV-TRPC6-GFP (TRPC6 overexpression) infected neurons treated with or without MK801 in response to glutamate (100 μM) application. Three neurons per experiment were analyzed. *n* = 3 independent experiments/group. ^*^P < 0.01. **(D)** Glutamate-evoked increase in [Ca^2+^]_*i*_ was significantly reduced in control (AdCMV-GFP) and AdCMV-TRPC6-GFP neurons after Na^+^ was removed and replaced by NMDG in the bath solution (Na^+^-free bath). The bar graph shows the peaks of F/F0 in AdCMV-GFP or AdCMV-TRPC6-GFP primary neurons treated with or without NMDG replacement in response to glutamate. Three neurons per experiment were used. *n* = 3 independent experiments/group. ^*^P < 0.01, ^#^*P* < 0.05.

To demonstrate whether TRPC6 could mediate Ca^2+^ influx and whether such an influx was dependent on AMPA channel receptors whose opening also increased the opening probability of NMDA receptor channels (see below), WT cortical neurons were infected for 4 h with AdCMV-TRPC6-GFP to overexpress TRPC6 and AdCMV-GFP as control (normal TRPC6 expression). Twenty-four hours after infection, neurons showed GFP (green) (Figure 5A, last column) to confirm the successful infection. Glutamate (100 μM) was applied to a bath containing infected neurons in the presence of the AMPA antagonist CNQX (10 μM) that could effectively block AMPA receptors on the glutamate-induced [Ca^2+^]_*i*_ elevation (Dixon and Copenhagen, 1992; Regan and Choi, 1994). Different from the control group where the intracellular Ca^2+^ level peaked at 60 s, [Ca^2+^]_*i*_ was enhanced in response to glutamate over the whole recording period (300 s) in TRPC6 overexpressed neurons. In the presence of CNQX, overexpression of TRPC6 still enhanced [Ca^2+^]_*i*_ in response to glutamate application, indicating TRPC6 could mediate Ca^2+^ influx in response to glutamate independent of AMPA receptors (Figure 5B).

### TRPC6 overexpression-induced [Ca^2+^]_*i*_ increase was attributed to NMDAR activation

To determine whether the glutamate-evoked Ca^2+^influx increase observed in TRPC6-infected neurons was attributable to NMDAR activation, we applied NMDAR antagonist MK-801. WT cortical neurons were infected with AdCMV-TRPC6-GFP to enhance TRPC6 expression and AdCMV-GFP for control. Glutamate (100 μM) was applied to a bath containing infected neurons in the presence of MK-801 (20 μM) which could effectively block NMDAR receptor mediated-, glutamate-induced [Ca^2+^]_*i*_ increases (Wong et al., 1986; Tasker et al., 1992). After glutamate application, [Ca^2+^]_*i*_ was evoked. TRPC6 overexpression significantly increased glutamate evoked [Ca^2+^]_*i*_ compared to the control but MK-801 reduced such an increase (Figure 5C). Thus, TRPC6 overexpression induced the glutamate evoked [Ca^2+^]_*i*_ elevation that is largely attributed to NMDAR activation.

### TRPC6 overexpression-mediated [Ca^2+^]_*i*_ increase was attributed to Na^+^ entry

Na^+^ influx regulates neuronal excitability and is responsible for the initiation and propagation of action potentials (Yu and Salter, 1998). NMDA receptor-mediated Ca^2+^ influx can be enhanced by raising intracellular Na^+^ ([Na^+^]_*i*_) or activating Na^+^-permeable channels (Taylor and Meldrum, 1995; Yu, 2006). To determine whether the activity of NMDAR may be regulated by TRPC6 via the actions of Na^+^ entering neurons, we examined glutamate-evoked Ca^2+^ levels in TRPC6 overexpressed neurons by completely eliminating Na^+^ in the culture medium. Na^+^ was replaced by the large membrane-impermeable cation N-methyl-D-glucamine (140 mM NMDG replacing 140 mM NaCl) in the bath solution such that the electrical charges and osmolality across the cell membrane would not be changed (Storozhevykh et al., 1998). Again, WT cortical neurons were infected with AdCMV-TRPC6-GFP to enhance TRPC6 expression and infected with AdCMV-GFP as control. After 24 h infection, [Ca^2+^]_*i*_ was significantly enhanced in TRPC6 overexpressed neurons compared to control group (GFP) in response to glutamate application. NMDG replacement significantly, but not completely, reduced such an increase in TRPC6-infected neurons (Figure 5D). Thus, the glutamate-evoked increase in [Ca^2+^]_*i*_ was significantly reduced in TRPC6 overexpressed neurons in Na^+^-free bath, suggesting that TRPC6 induced [Ca^2+^]_*i*_ increase was, at least partially, attributed to Na^+^ entry to the neurons.

### OGD-induced [Ca^2+^]_*i*_ increase may be attributed to voltage-sensitive Na^+^ channels and AMPA receptor channels

**Figure 6:**
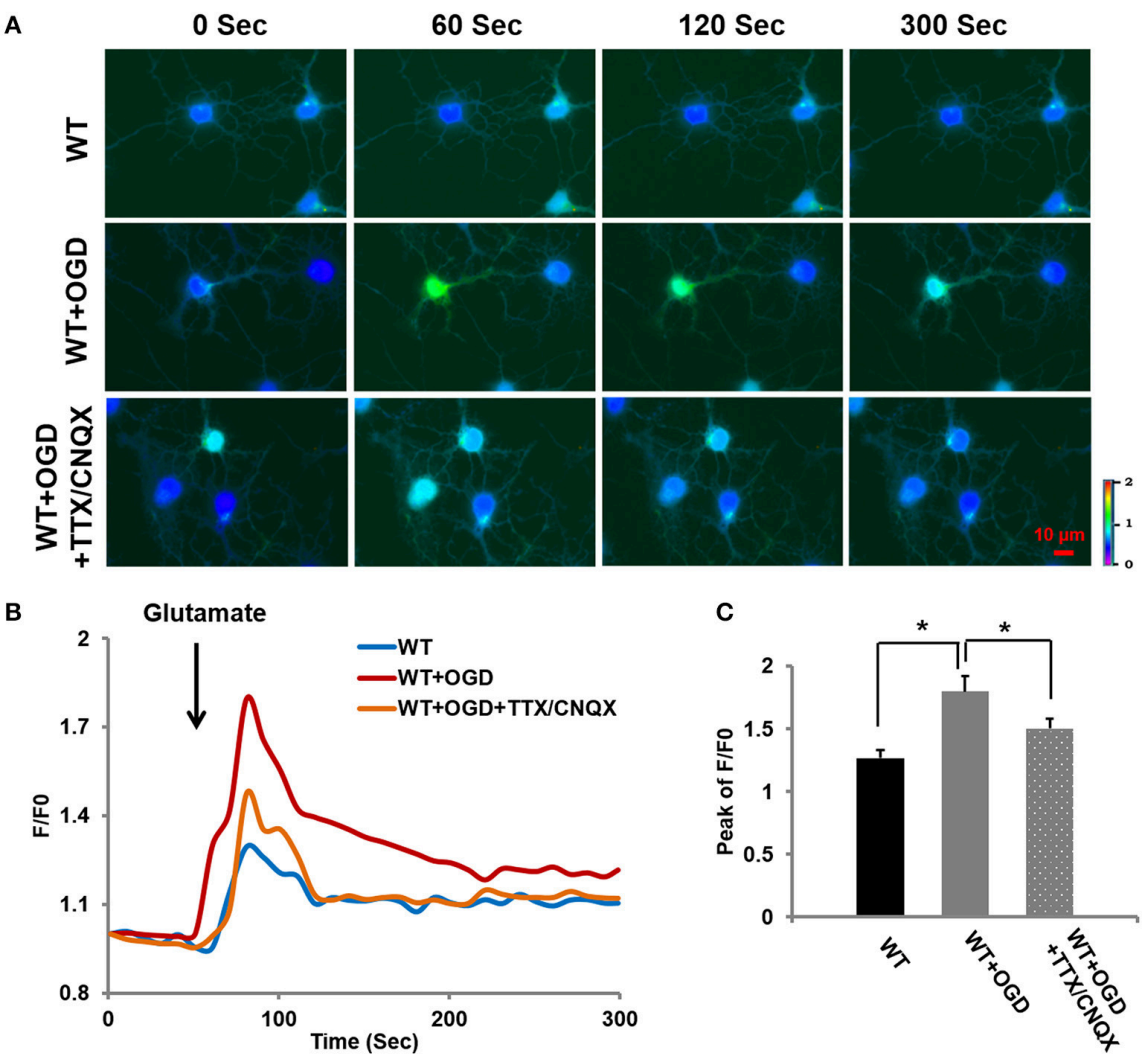
**OGD-induced [Ca^**2+**^]i increase may be attributed to voltage-sensitive Na^**+**^ channels and AMPA receptor channels. (A)** Ca^2+^ influx in OGD-pretreated WT primary neurons in response to glutamate (100 μM) application in the bath containing TTX (0.1 μM) and CNQX (10 μM) to block voltage-sensitive channels and AMPA receptors. **(B)** Traces show the time course of the intracellular [Ca^2+^]i. **(C)** The bar graph shows the peaks of F/F0 traces. Ten neurons per experiment were analyzed. *n* = 3 independent experiments/group. ^*^P < 0.01.

As shown in Figure 4, TRPC6 mediated Ca^2+^ entry following OGD. We further assessed whether OGD-induced increase in [Ca^2+^]_*i*_ was also mediated by other channels, e.g., voltage-sensitive Na^+^ channels and AMPA receptor mediated channels, voltage-sensitive Na^+^ blocker Tetrodotoxin (TTX, 0.1 μM) was applied to block voltage-sensitive Na^+^ channels in the presence of AMPA receptor blocker CNQX (10 μM) (Tasker et al., 1992; Glazner et al., 2000; Tu et al., 2009). The Ca^2+^ influx in response to glutamate application was measured in WT cortical neurons with OGD pretreatment in presence of TTX and CNQX, along with WT cortical neurons with or without OGD. OGD induced [Ca^2+^]_*i*_ increase was significantly reduced, *but not completely eliminated* by TTX/CNQX in neurons compared to the control neurons (WT) (Figure 6). In addition to TRPC6 voltage-insensitive channels that contributed OGD-enhanced Ca^2+^ influx, voltage-sensitive Na^+^ channels or/and AMPA receptors may contribute to OGD-enhanced Ca^2+^ influx. This indicates that Na^+^ entry via TRPC6, voltage-sensitive Na^+^ channels and AMPA receptor channels all play a role in facilitating depolarization-induced NMDAR activation during OGD.

## Discussion

For decades, Ca^2+^ overload has been considered as one of the major mechanisms underlying ischemia-induced neuronal death. Blockers of NMDA receptors were used to reduce Ca^2+^ influx and showed protective effects on the brain damages in animal stoke models. Unfortunately, multiple clinical trials of glutamate antagonists failed to show effective neuroprotection and had serious side effects in stroke patients (Roth and Liesz, 2016). There is an urgent need to develop new, effective alternative targets for treatment. Here, the present study reports that deletion of TRPC6 protects against cerebral I/R-induced brain damage *in vivo* and OGD- and excitatory neurotoxin-induced cell death *in vitro*. This protection is mediated, in part, through the TRPC6 mediated NMDA receptors. Specifically, TRPC6 expression was increased in the brain tissues in the I/R animal model and OGD-treated cortical neurons in culture. In addition, deletion of TRPC6 protected I/R-induced brain damage and OGD-induced neuronal death. Furthermore, deletion of TRPC6 could also protect neurons against excitatory neurotoxin (glutamate or NMDA)-induced cell death. To determine the cellular and molecular mechanism for OGD-induced neuronal death, OGD was found to increase, but deletion of TRPC6 reduced glutamate-evoked Ca^2+^ entry. TRPC6 overexpression increased glutamate-evoked Ca^2+^ entry in the presence of the AMPA blocker, indicating TRPC6-increased Ca^2+^ entry could be independent of AMPA receptor channels. In the presence of the NMDA receptor antagonist MK801, the increased glutamate-evoked Ca^2+^ entry in TRPC6 overexpressed neurons was largely reduced, indicating that NMDA receptors were involved. To assess whether Na^+^ contributed to the TRPC6-enhaced Ca^2+^ influx, we found that glutamate-evoked Ca^2+^ entry in TRPC6 overexpressed neurons was largely reduced in Na^+^-free bath, indicating that Na^+^ entry was required for TRPC6-mediated Ca^2+^ influx. Finally, we found that glutamate-evoked Ca^2+^ entry was reduced *but not completely* abolished in OGD-treated WT neurons after blockade of voltage-sensitive Na^+^ channels and AMPA receptor channels. We postulate that TRPC6 channels, as well as other channels (e.g., voltage-sensitive Na^+^ channels), may contribute to TRPC6-mediated Ca^2+^ influx, at least in part, through NMDA receptor channels. Therefore, the present data have demonstrated that overexpression of TRPC6 may contribute to neuronal death partially through Na^+^ entry, leading to activation of NMDA receptors, thus causing an overload of Ca^2+^.

The present results are consistent with several findings about TRPC6 in different tissues and organs. Previously, it has been demonstrated that hypoxia could drive glioblastoma multiforme cells into a more aggressive and malignant state through Notch1-induced TRPC6 overexpression which in turn activated the calcineurin-nuclear factor of activated T-cell (NFAT) pathway and increased Ca^2+^ influx (Chigurapati et al., 2010). Later, TRPC6^−/−^ mice were found to be resistant to I/R-induced edema in the lung (Weissmann et al., 2012). TRPC6-deficient mice attenuated I/R -induced Ca^2+^ influx, cellular shape changes, and barrier dysfunction in the lung. Along this line, it has been further reported that activation of TRPC6-dependent Ca^2+^ signaling facilitated endotoxin-induced lung vascular permeability and inflammation (Tauseef et al., 2012). Moreover, TRPC6 deletion in mice increased resistance to endotoxin-induced barrier dysfunction and inflammation, and protected against sepsis-induced lethality. In the kidney, acquired glomerular diseases were associated with increased expression levels of TRPC6. It has been reported that overexpression of TRPC6 in podocytes was sufficient to cause a kidney disease consistent with focal and segmental glomerulosclerosis (Krall et al., 2010). Though the above studies were from non-neural tissues, they all indicated that overexpression or activation of TRPC6 was involved in disease-related damages in tissues and organs.

Consistent with the present finding, TRPC6 expression levels in the retinal ganglion cells were increased 24 h after I/R in rats (Wang et al., 2010). Similarly, TPRC6 levels were increased in patients with glaucoma (Chen et al., 2013). Different from the present findings, however, activation of TRPC6 before ischemia has been reported to have neuroprotective effects on retinal ganglion cells (Wang et al., 2010). In contrast, TRPC6 expression levels were decreased in the brain after I/R and activation of TRPC6 had protective effects on ischemic brain damage in rats (Du et al., 2010). Furthermore, TRPC6 activator Hyperforin and Resveratrol has been shown to suppress MCAO-induced brain damage via inhibition of TRPC6 degradation in rats (Lin et al., 2013a,b). Thus far, the exact reasons for these discrepancies among these studies are not known. Possibly, different animal models, tissue types, and approaches contributed to these discrepancies. Whereas other studies used rat models, we used the TRPC6^−/−^ mouse model. Further investigation is needed to address these discrepancies.

It should be mentioned that TRPC1 or TRPC3 are also widely expressed in the nervous system and they have structural similarities with TRPC6 (Riccio et al., 2002). In addition to TRPC6, the expression level of TPRC1 in the brain tissue was measured after I/R in WT mice and the TRPC3 expression level in the primary cultured neurons following OGD in the early stage of the present study. In contrast to TRPC6 expression as shown in Figure 1, expression of TRPC1 was not increased in the ipsilateral cortex after I/R and TRPC3 expression was not increased in OGD-treated cortical neurons (unpublished observations). These initial observations lead to the selection of TRPC6, rather than TRPC1 and TRPC3 in this study. Whether TRPC1 and TRPC3 may be involved in I/R-induced brain damage should be studied in the future.

**Figure 7:**
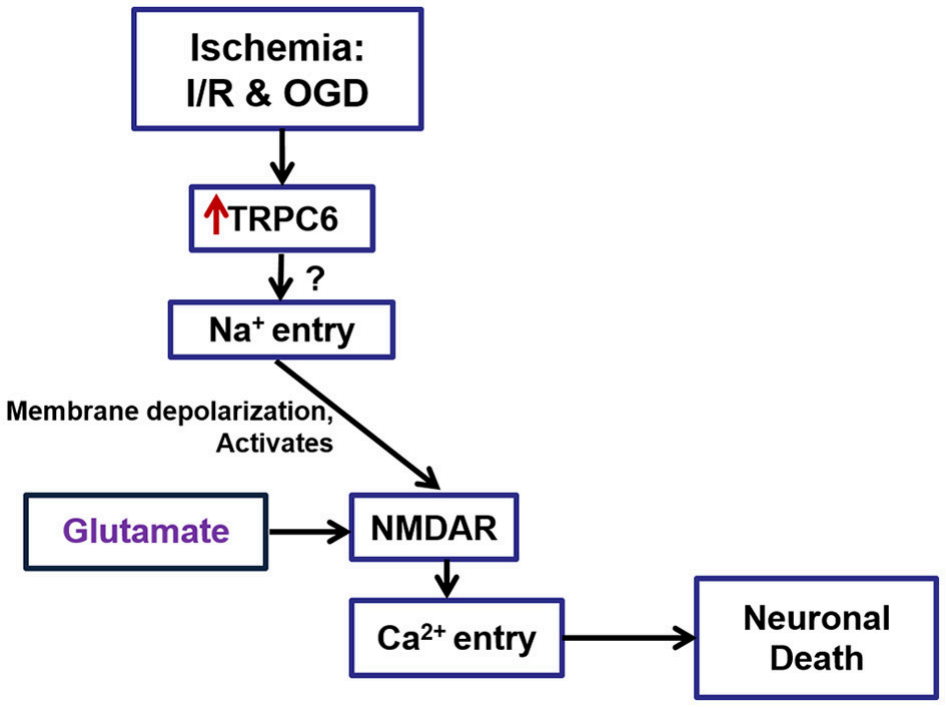
**A schematic drawing to show the role of TRPC6 in I/R- and ODG-induced Ca2+ influx via regulation of NMDARs**. TRPC6 expression was increased in neurons following I/R in mice brain and OGD in primary cortical neurons. TRPC6 up-regulation following I/R and OGD induced cortical neuronal death may in part through Na^+^ entry, leading to over-activation of NMDA receptors, and causing an overload of Ca2^+^. Deletion of TRPC6 protects against cerebral ischemia-induced brain damage *in vivo* and OGD- and excitatory neurotoxin-induced cell death *in vitro*. This protection is mediated partially through TRPC6-mediated NMDA pathways.

In summary, the present studies have demonstrated that TRPC6 overexpression after I/R and following OGD treatment may contribute to brain damage and to OGD- and neurotoxicity-induced cell death. Activation of TRPC6 channels may increase Ca^2+^ influx through NMDAR, which may lead to cell death (Figure 7). Since TRPC6 channels are voltage-independent and are not involved in the major neurotransmission, pharmacologic interventions targeting at TRPC6 may potentially have fewer side effects than direct blockade of NMDAR. Though this study has provided a potential mechanism for the TRPC6 deletion protection against Ca^2+^ -induced neurotoxicity through NMDAR, the present work has not yet determined whether blockade of TRPC6 may have a greater protective effect than directly blocking NMDAR. Such an important question needs to be addressed in future studies.

## Ethics statement

This study was carried out in accordance with the recommendations of The Guide for the Care and Use of Laboratory Animals, 8th edition. The protocol was approved by the UCF IACUC.

## Author contributions

JC, Data collection, data analysis and interpretation, manuscript writing; ZL, Data collection, data analysis, manuscript editing; JH, Concept contribution, manuscript editing; QC, Concept contribution and Manuscript editing; LC, Data analysis; RW, Concept contribution to the manuscript, manuscript editing; SC, Design of the work, provide Figures 1A,B, 2A of the article; ZC Design of the work, manuscript writing and editing, final approval of the version submitted.

### Conflict of interest statement

The authors declare that the research was conducted in the absence of any commercial or financial relationships that could be construed as a potential conflict of interest.
